# Neural correlates of sense of agency in motor control: A neuroimaging meta-analysis

**DOI:** 10.1371/journal.pone.0234321

**Published:** 2020-06-05

**Authors:** Giuseppe A. Zito, Roland Wiest, Selma Aybek

**Affiliations:** 1 Department of Neurology, Inselspital, Bern University Hospital, University of Bern, Bern, Switzerland; 2 Support Centre for Advanced Neuroimaging (SCAN), University Institute for Diagnostic and Interventional Neuroradiology, Bern University Hospital Inselspital, University of Bern, Bern, Switzerland; Universidade Estadual Paulista Julio de Mesquita Filho, BRAZIL

## Abstract

The sense of agency (SoA) refers to the perception that an action is the consequence of one’s own intention. Studies exploring the SoA with neuroimaging techniques summarized the available data and confirmed a role of fronto-parietal areas and subcortical structures. However, these studies focused on specific regions of interest. We thus conducted a whole-brain meta-analysis to verify which regions emerge as significant for the SoA, specifically during motor execution. We performed a systematic search on PubMed, PsycINFO and Cochrane databases with the following inclusion criteria: studies investigating SoA with a visuo-motor task by means of neuroimaging in healthy subjects. We performed a quantitative, whole-brain, meta-analysis of neural correlates of the SoA based on the activation likelihood estimation. Of the 785 articles identified by our search, 22 studies met our inclusion criteria (169 foci, 295 subjects for decreased agency, and 58 foci, 165 subjects for normal agency). Neural correlates of decreased agency were the bilateral temporo-parietal junction (MNI: 50,-54,14; -44,-52,42; -48,-56,8). Normal agency showed no significant clusters of activation. This meta-analysis confirmed the key role of areas responsible for decreased SoA during motor control, whereas normal agency did not show a specific neural signature. This study sets the ground for future regions-of-interest analyses of neural correlates of SoA, as well as potential neuromodulation studies, which might be relevant in medical conditions presenting with abnormal SoA.

## Introduction

The sense of agency (SoA) is an important aspect of human self-consciousness, that allows us to distinguish between self-generated actions or thoughts and external ones [1, 2]. This cognitive process is fundamental for our interaction with the external world [3], and an impairment in the SoA has been linked with several neuropsychiatric conditions, such as schizophrenia [4, 5], alien hand syndrome [6], or functional neurological disorders [7, 8].

The SoA can be explained by the “comparator” model, a theory initially developed to account for motor learning and control [9], and then later expanded to agency processing [10, 11]. According to this model, two neural processes contribute to the formation of agency, the forward (feedforward) and the inverse (feedback) processes. The first one provides the motor commands necessary to perform an action, and makes predictions about the behavior of the motor system [12, 13]. The second one provides the actual sensory consequence of the performed action. The predictions are of key importance, because they are compared with the actual sensory feedback from the movement. In case of perfect match, we feel that we are in control of our actions. Conversely, in case of mismatch, a certain degree of incongruency is perceived, and we feel that we are not the agent of the action [14]. This is typically the case for externally generated or passive movements, which are not associated with any motor intention and cannot be predicted by the forward process [12, 15]. The comparator model fits well with a non-conceptual feeling of agency, where discrepancies between actual and intended movements are unconsciously detected. However, it does not account for a higher order, conceptual judgement of agency, where agency assignments to the self or the other are made [16]. Indeed, it has been proposed that feeling and judgement of agency are two distinct neural processes, whose interaction gives rise to the SoA [17]. More specifically, if the feeling of agency has been defined as an unconscious ongoing flow of action processing, where actions are simply tagged as self-caused or not [17], the judgement of agency is its conscious counterpart, where several multidimensional factors, including expectations of action and beliefs of being the agent of the movement, are processed [16, 18].

Studies over the last 20 years have investigated the SoA, in both healthy subjects and patients [19]. Well-established paradigms for this consist of computer-based games, where participants first perform a motor task, and then are asked to evaluate their sense of control over their actions. In such games, the SoA can be artificially distorted by introducing a delay [20–24], or by manipulating the congruency of the visual feedback [21, 25], thus triggering the feeling that we are not in control of the executed movement and, possibly, that the movement is performed by someone else.

Neuroimaging studies have mainly focused on the feeling of agency, and have found that the SoA involves areas devoted to the motor system (ventral premotor cortex [26, 27], supplementary motor areas–SMA [20, 26, 28–31] and pre-SMA [21, 31–37]–cerebellum [20, 26, 28, 30–32, 34, 37–41]), as well as to cognition and multimodal information processing, such as the dorsolateral prefrontal cortex [3, 33, 39, 40, 42], the posterior parietal cortex (PPC) [30, 31, 36, 38, 39, 42] and the insula [20, 25, 32, 36–40]. In particular, the PPC has been identified as a key area for agency processing, as it plays an important role in mismatch detection between intended and actual consequences of an action [30, 33, 36, 40].

The literature on the SoA can be broadly divided into two kinds of experiments: the study of brain activations in response to *decreased* sense of control, occurring when the SoA is disrupted by artificially impairing the congruence between movement and visual feedback, and in response to *normal* sense of control [1], when no manipulation is present. Different brain activations have been identified in response to these processes, but no clear discrimination between these two networks has been established yet.

Previous meta-analyses on brain activation in response to manipulation of SoA have focused on specific areas [21, 43], or on the SoA from a general point of view [1], and not specifically during motor tasks. Indeed it has been shown that SoA mainly arises when an action is performed [44], and this process should be thus investigated in relation to motion. This paper aims to shed light on the brain networks of reduced and normal agency, during motor control, and to precisely locate these networks on the cortex, under the assumption that the two processes activate different brain regions. To this end, we systematically reviewed the literature investigating the SoA over the last 20 years, and conducted a quantitative, whole-brain, meta-analysis, following the Preferred Reporting Items for Systematic review and Meta-Analysis protocols (PRISMA-P) guidelines [45], of the neural correlates of the SoA, with specific focus on studies manipulating the sense of control over a behavioral motor task.

## Materials and methods

This study was carried out in accordance to the latest version of the Declaration of Helsinki. As this study analyzed data already published, no informed consent was needed.

### Literature search

We searched the PubMed, PsycINFO, and Cochrane databases between January 1999 and January 2019. We did not include earlier studies because the definition of the SoA, as well as the quality of the neuroimaging techniques, may have been different earlier, and could not guarantee the inclusion of studies with comparable results. Search terms were: "magnetic resonance imaging" [All fields] OR Fmri [All fields] OR PET [All Fields] OR "positron emission tomography" [All Fields] AND “agency” [All Fields] AND "humans" [MeSH Terms]. Moreover, we used the “related articles” function of the PubMed database to identify additional papers. We also manually searched reference lists of articles identified by our search. The inclusion criteria were: 1. trials involving healthy adult (18 years old or older) human subjects, studies engaging their participants in a behavioral motor task where the visual feedback was artificially manipulated in the way that it was not always coherent with the motor intention, 2. studies using neuroimaging techniques, 3. studies published in peer-reviewed journals, 4. studies in English. Exclusion criteria were: 1. studies in which the brain areas where not clearly identified by standard Talairach (TAL) or Montreal Neurological Institute (MNI) coordinates, 2. studies where no significant clusters of activation were found.

We screened the titles and abstracts yielded by the search against the inclusion criteria and obtained full reports for all papers that appeared to meet the inclusion criteria. The papers were then examined in more details to see whether they actually met the inclusion criteria. None of the authors was blind to the journal titles or to the study authors or institutions.

### Risk of bias assessment and quality of evidence

The selected papers were reviewed for risk of bias using the Cochrane Risk of Bias tool (RoB 2.0) [46], and categorized as “low risk”, “some concerns” or “high risk” based on the following items of the RoB: randomization, deviation from intervention, missing data, and measurement of the outcome. We excluded the “risk due to selection of the reported results” from the analysis, as the investigated studies reported only one main outcome, i.e., the brain activation patterns in response to manipulation of agency.

### Meta-analysis

The main outcomes we extracted were the MNI or TAL coordinates of the activation peaks in response to experience of agency, as well as basic demographic data of the investigated samples. We defined negative agency as the experience of reduced motor control, and positive agency as its exact opposite, i.e., the experience of normal control. We performed two separate analyses, for negative and positive agency, respectively. Negative agency was identified by the contrasts negative VS positive agency, whereas positive agency was identified by the contrasts positive VS negative agency.

The analysis of the activation peaks was performed using activation likelihood estimation (ALE) [47, 48], implemented in GingerALE 3.0.2. The foci (coordinates of the maximum activation) identified from each study were first converted into standard MNI space, if needed, and then modelled as the peaks of a 3D Gaussian probability distribution. ALE scores were calculated on a voxel-by-voxel basis, by taking the union of the individual “modelled activation” maps, i.e., the maps of active brain areas resulting from each single study. Statistical significance was assessed with a voxel-level threshold of p < 0.001, and a cluster-forming threshold of p < 0.05 corrected with Family-Wise Error (FWE), and 5000 permutation tests to correct for multiple comparisons [49]. Each ALE map was finally overlaid onto an anatomical template obtained by normalizing the International Consortium for Brain Mapping (ICBM) template to the MNI space.

## Results

### Literature search

**Fig 1:**
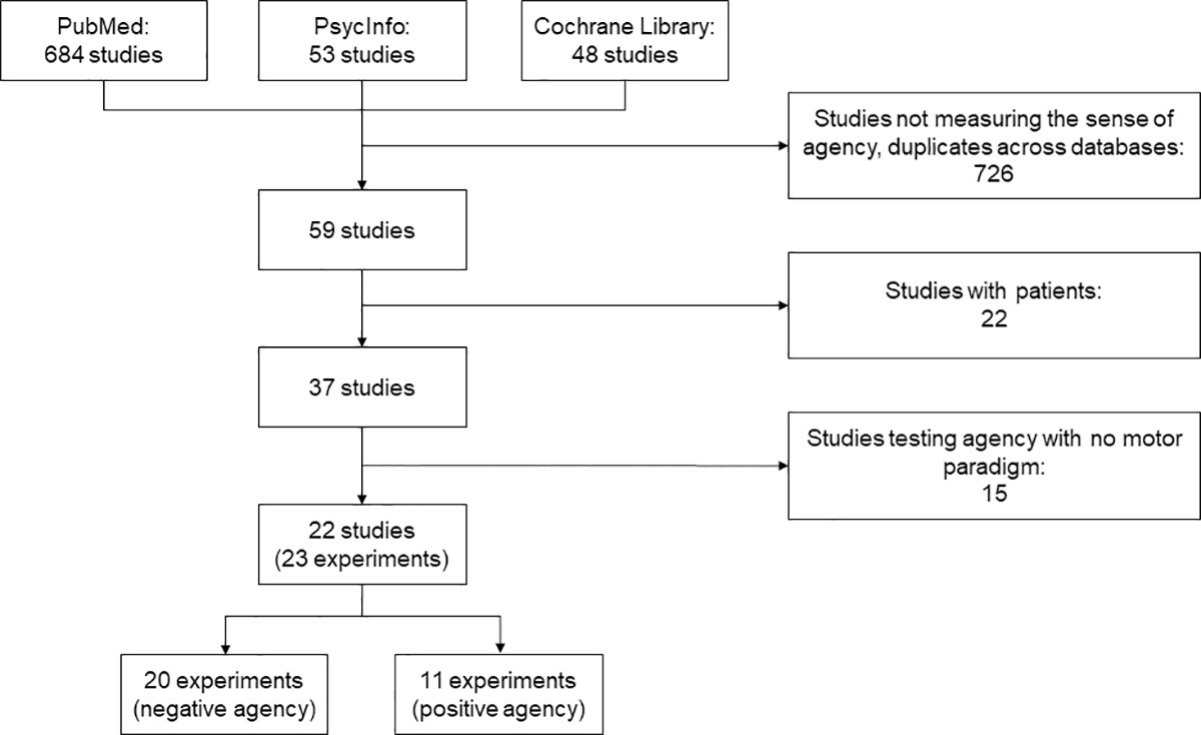
Flow chart of the literature search.

**Fig 2:**
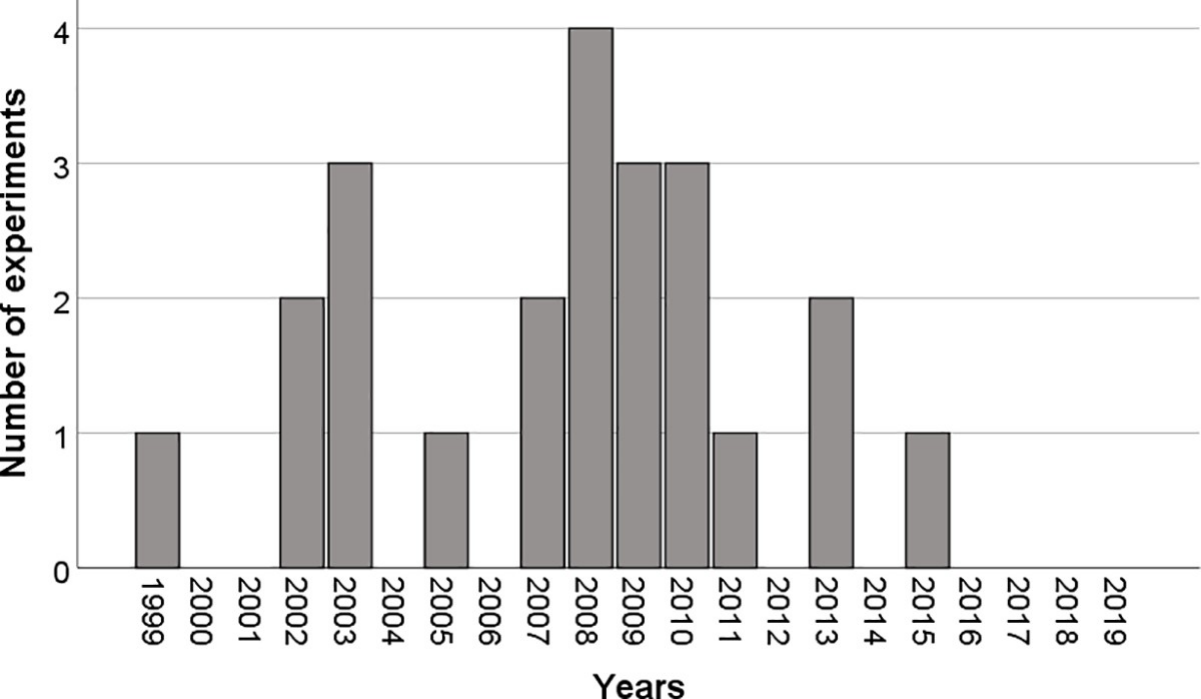
Distribution of experiments investigating the sense of agency over the last 20 years.

The literature search yielded a total of 684 studies from the PubMed database, 53 from PsycInfo, and 48 from the Cochrane Library. We then filtered out the studies not measuring the SoA, as well as duplicates across databases. Out of the 59 remaining studies, we further excluded 22 studies with patients (e.g., alien hand syndrome, functional neurological disorders, major depression, schizophrenia), and 15 studies where the sense of agency was not tested with a motor paradigm and an artificial manipulation of the visual feedback. The final selection was 22 studies (23 experiments), of which 19 were performed with fMRI techniques and 3 with PET (Fig 1, Table 1). The distribution of the experiments over the years is shown in Fig 2.

**Table 1:** Results of literature search.

Study	Subjects	Imaging method	Task	Type of agency	Contrast	Coordinates (x,y,z)			Coordinate space
Agnew et al., 2008 [28]	20	fMRI	finger tap	negative	passive VS active finger tap	48	-30	26	MNI
						32	-36	62	
						-52	-22	50	
						-46	-28	24	
				positive	active VS passive finger tapping	4	-50	-20	
Balslev et al., 2008 [51]	15	fMRI	cursor movement	negative	asynchronous VS synchronous stimulation	54	-42	33	MNI
						-42	-51	45	
						-54	-48	27	
						51	-24	-12	
David et al., 2007 [38]	14	fMRI	joystick movement	negative	incongruent VS congruent feedback	40	44	22	MNI
						-2	-64	48	
						-40	-48	42	
						2	12	62	
						-42	-2	56	
						52	-34	-10	
						40	50	12	
						60	-42	38	
						34	4	60	
						-40	-40	58	
						-44	-66	10	
Decety et al., 2002 [52]	18	PET	hand manipulation of 3D geometrical objects	positive	imitation of the self by the other VS self action	54	-52	40	MNI
						14	30	40	
						0	44	38	
						46	20	30	
						14	12	10	
						52	22	8	
						-6	12	56	
						-12	-50	40	
Farrer et al., 2002 [25]	12	fMRI	joystick movement	negative	other attribution VS self-attribution	44	-58	32	MNI
						-48	-52	40	
						-6	-58	50	
						2	-50	44	
						-38	28	48	
				positive	self-attribution VS other attribution	40	8	2	
						-36	-2	2	
Farrer et al., 2003 [32]	8	PET	joystick movement	negative	25° deviation VS 0°, in conjunction with 50° VS 25° and other-controlled VS 50°	56	-56	36	MNI
						-64	-58	32	
						0	14	54	
						50	10	58	
						12	30	42	
Farrer et al., 2008 (Experiment 1) [33]	15	fMRI	manual peg removal task: removing as many golf pegs as possible from a grid within a time limit	negative	delay VS no delay	44	-54	38	MNI
						-40	-58	36	
						-48	-38	-54	
						-22	52	32	
						28	52	40	
						-30	24	-12	
						-10	26	64	
						-34	-20	66	
						-14	-42	6	
Farrer et al., 2008 (Experiment 2) [33]	18	fMRI	alternate index and middle finger movement	negative	perturbed visual feedback VS unperturbed visual feedback	58	-46	48	MNI
						44	-50	60	
						-48	-46	56	
						-48	28	30	
						-44	22	36	
						28	54	-2	
						46	30	42	
						-54	18	20	
						38	50	-2	
Fink et al., 1999 [42]	23	PET	Luria’s bimanual coordination task: open and close the two hands	negative	out-of-phase VS in-phase hand movement	44	22	32	TAL
						-50	18	48	
						50	-54	38	
						-52	-44	42	
						-30	-66	-42	
Fukushima et al., 2013 [40]	17	fMRI	key press	negative	no agency judgement VS agency judgement	48	-25	62	MNI
						-8	0	31	
						-15	-32	-15	
				positive	agency judgement VS no agency judgement	10	-42	20	
						38	-91	-8	
						-22	-84	-33	
						-5	-56	48	
						-33	-60	27	
						-36	21	41	
						38	18	13	
Kontaris et al., 2009 [34]	11	fMRI	hand actions, such as fist closing and finger extension	negative	incompatible VS compatible condition	57	-55	19	TAL
						60	-46	-5	
						45	12	22	
						48	17	2	
						9	-55	34	
						42	5	34	
						0	38	43	
						-54	-52	16	
						-6	8	55	
						-45	11	31	
						-42	-46	-8	
						-42	20	2	
				positive	compatible VS incompatible condition	24	-85	1	
						24	-76	16	
Kühn et al., 2013 [35]	17	fMRI	key press	positive	active VS passive condition	-11	-8	74	MNI
Leube et al., 2003a [50]	6	fMRI	open and close the hand	negative	displaying other’s hand VS own hand	12	-87	-6	MNI
						9	39	24	
						9	-87	27	
				positive	displaying own hand VS other’s hand	39	-75	-6	
						42	-60	-9	
						36	-72	24	
						-30	-81	21	
						45	9	24	
						27	-51	63	
Leube et al., 2003b [20]	18	fMRI	open and close the hand	negative	delay VS baseline	33	21	-6	MNI
						54	12	9	
						48	6	36	
						51	-69	3	
						24	-93	15	
						-24	-96	9	
						-33	21	-6	
						-51	6	36	
						-3	18	48	
						-54	-24	27	
						-33	-36	63	
						-15	12	-6	
Matsuzawa et al., 2005 [26]	6	fMRI	key press	negative	delayed VS synchronous	10	-57	-6	TAL
						36	-42	44	
						-2	-75	9	
						-40	-15	56	
						-10	-4	70	
						28	-1	61	
						57	12	3	
				positive	synchronous VS delayed	26	-53	-19	
						38	-50	58	
						-38	-17	58	
						4	3	68	
						32	1	59	
						57	10	5	
Miele et al., 2011 [21]	11	fMRI	cursor movement	negative	conditions with turbulence VS conditions without turbulence	56	-34	26	MNI
						68	-34	18	
						58	-34	14	
						48	-62	4	
						26	-16	68	
						14	-28	66	
						12	-8	68	
						-48	-34	18	
						-40	-48	24	
						-46	-66	8	
						-46	-56	10	
						-60	-64	6	
						-16	-38	70	
						-34	-42	70	
						-28	-58	64	
Nahab et al., 2010 [36]	20	fMRI	finger movement with virtual hand (cyber glove)	negative	coherent VS incoherent hand movement	56	-50	12	TAL
						52	-46	18	
						20	8	56	
						62	-50	6	
						22	8	62	
						40	-46	14	
						58	-52	36	
						-50	-52	44	
						-38	-56	-40	
						32	2	54	
						-32	20	2	
						8	-56	42	
						-38	46	24	
						34	-58	36	
						-34	16	12	
						21	20	5	
						50	-56	24	
						40	26	42	
						38	10	32	
						52	-40	-4	
						8	8	54	
						46	-62	38	
						46	-56	50	
						-44	-50	38	
						44	8	54	
						-14	-70	-28	
						-46	40	18	
Renes et al., 2015 [53]	23	fMRI	key press to move a square on a screen	positive	agency over the square movement VS no agency	-52	-68	32	MNI
						-20	52	40	
						20	36	52	
						8	64	4	
Schnell et al., 2007 [30]	15	fMRI	bimanual joystick movement	negative	monitor of incongruence VS control condition	45	20	40	TAL
						50	36	15	
						39	20	49	
						24	59	5	
						27	65	11	
						-3	20	40	
						-3	34	37	
						3	40	39	
						62	-51	22	
						53	-45	24	
						-59	-51	36	
						-50	-53	47	
						9	-71	39	
						62	-24	-14	
						65	-36	-11	
						-45	-58	8	
						0	-25	21	
						-15	-77	-31	
				positive	control condition VS monitor of incongruence	62	4	25	
						-56	1	22	
						-3	61	-6	
						-36	-21	54	
						33	-29	54	
						-48	-18	42	
						33	-30	48	
						50	3	-5	
						-45	-6	-5	
						-65	-26	7	
						-15	-55	6	
						27	5	-8	
						30	-15	-2	
						-12	-60	-25	
						18	-54	-25	
Spengler et al., 2009a [27]	17	fMRI	finger movement	negative	increasing activation with increasing sensory-motor discrepancy	49	-52	18	TAL
						-47	-55	12	
Spengler et al., 2009b [41]	18	fMRI	key press	negative	increasing activation with increasing sensory-motor discrepancy	49	-52	18	TAL
						-47	-55	12	
						-35	17	24	
Tsakiris et al., 2010 [37]	20	fMRI	finger movement	negative	asynchronous VS synchronous stimulation	40	-58	26	MNI
						52	-38	38	
						-38	20	2	
						-16	-84	-26	
						-12	-62	-38	
						40	52	14	
						24	48	-14	
						50	-46	-2	
						60	20	6	
				positive	synchronous VS asynchronous stimulation	-22	-54	-24	
						24	-40	54	
						38	-42	58	
						12	-48	-20	
						-44	-18	18	
						24	-72	36	
Yomogida et al., 2010 [31]	24	fMRI	joystick movement	negative	agency violation VS sensory match violation	-22	-66	-12	MNI
						-6	-4	52	
						6	8	60	
						30	-72	24	
						52	-58	12	
						42	-72	12	

20 experiments (169 foci, 295 subjects) were identified for negative agency, and 11 experiments (58 foci, 165 subjects) for positive agency.

### Risk of bias assessment

The investigation of the RoB revealed that two studies raised some concerns with regards to randomization bias (no information on randomization of the task repetitions) [34, 42], all studies were considered at low risk with regards to deviations from intended protocols (staff aware of the manipulation, but this was likely to have had no influence on the analysis of the results), bias due to missing data (data from all or nearly all participants were analyzed), and outcome assessment (the assessors were not blinded to the type of outcomes, but this was likely to have had no influence on the results).

### Summary of results of the meta-analysis

20 experiments explored neural correlates of negative agency (mean±SD of sample size = 15±5 subjects/experiment), of which six did not report complete demographic information of their sample [20, 25, 27, 31, 34, 50]. 146 male and 149 female subjects, 292 right handed, with age between 18 and 63, were tested. 11 experiments (sample size = 15±6 subjects/experiment) explored neural correlates of positive agency, of which three did not report complete demographic information of their sample [25, 34, 50]. 93 male and 72 female subjects, 163 right handed, with age between 18 and 50, were tested. Eight studies were conducted in Europe, three in the United States, and two in Japan. Nine studies did not report place of data collection.

Overall, we extracted 169 foci from 295 subjects for negative agency, and 58 foci from 165 subjects for positive agency.

**Fig 3:**
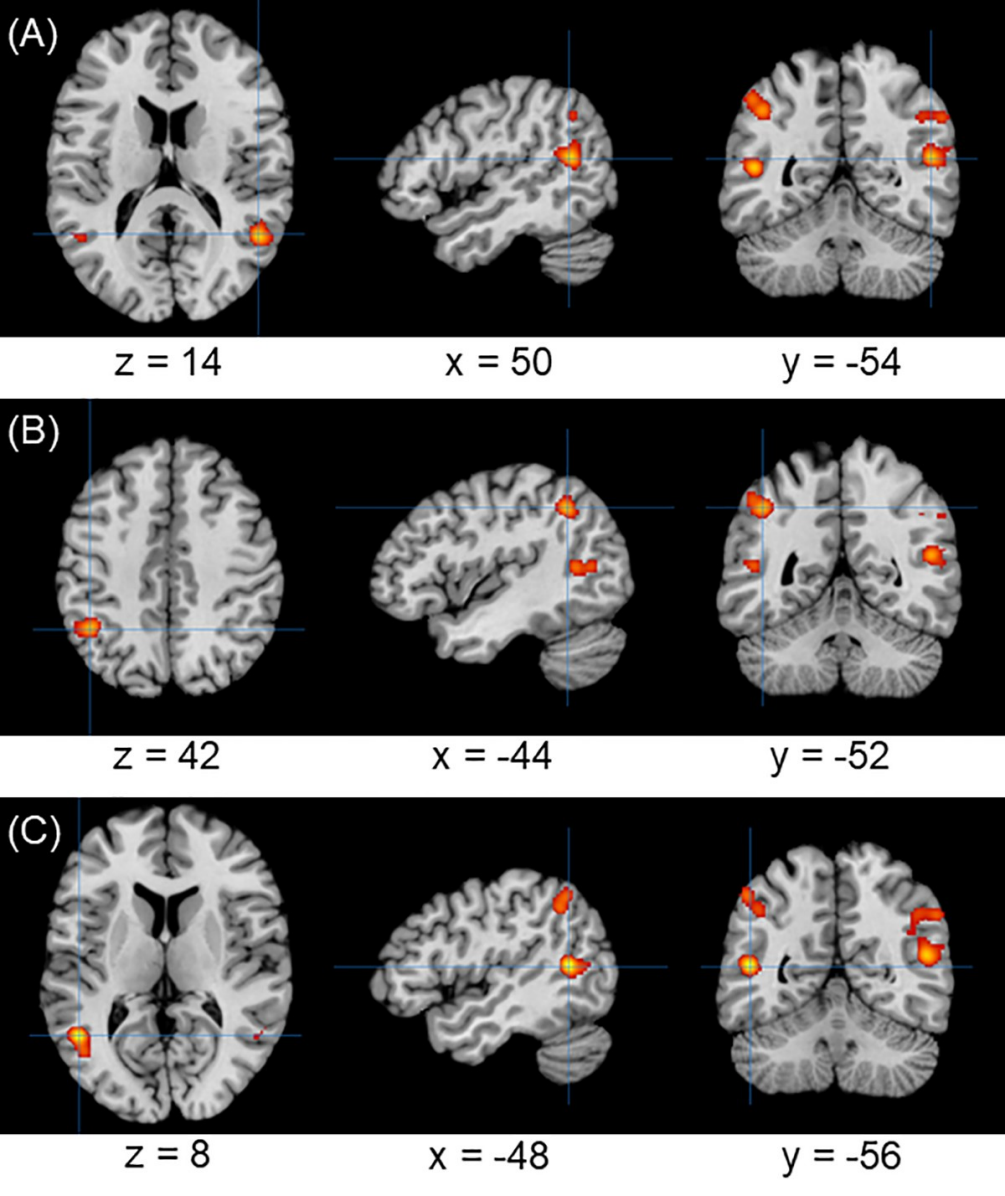
Results of the meta-analysis. Main brain areas showing hyper-activation patterns in response to negative agency. (A) cluster with peak on the right superior temporal gyrus. (B) cluster with peak on the left inferior parietal lobule. (C) cluster with peak on the left middle temporal gyrus. All clusters are FWE-corrected with p-value < 0.05.

The results of the meta-analysis evidenced three significant clusters of activation for negative agency, in the right superior temporal gyrus (Fig 3A), left inferior parietal lobule (Fig 3B), and left middle temporal gyrus, respectively (Fig 3C). Positive agency showed no significant clusters. Detailed results are shown in Table 2.

**Table 2:** Results of the meta-analysis.

Type of agency	Brain region	Cluster size (mm^3^)	Peak MNI Coordinates (x,y,z)			Peak ALE value
negative	Right superior temporal gyrus (63.3%) Right middle temporal gyrus (34.1%) Right angular gyrus (2.3%)	3320	50	-54	14	0.021*
	Left inferior parietal lobule (100%)	1888	-44	-52	42	0.020*
	Left middle temporal gyrus (58.3%) Left superior temporal gyrus (25%) Left middle occipital gyrus (16.7%)	1640	-48	-56	8	0.025*

All ALE values are significant at a FWE-corrected p-value < 0.05.

## Discussion

We performed a quantitative meta-analysis of 22 studies investigating the SoA by means of neuroimaging techniques, with the aim of precisely identify the location of the neural network of negative and positive agency for motor control. Our results showed an involvement of the bilateral temporo-parietal areas for processing of negative agency, whereas no clear areas devoted to positive agency were found.

### Negative agency

According to the comparator model of agency [9, 10], the brain area responsible for the comparison between the expected and actual motor outcome lays in the PPC, at the junction with the temporal lobe, a region often identified as temporo-parietal junction (TPJ) [1]. This area represents a crucial step in the comparator model, and it has been hypothesized that the activation of the TPJ is the neural signature of the lack of agency [1]. This is due to the fact that, when the action does not match its prediction, a compensatory movement for the unexpected outcome is often needed, and the trigger for this may come from the TPJ.

Interestingly, the coordinates of the mean peak of activity of the area identified as TPJ has not been consistent in the literature, with studies reporting activation in the angular gyrus [25, 27, 30–34, 36–38, 41, 42, 51], as well as in the middle temporal gyrus [21, 27, 30, 38, 41], the parietal operculum [21, 28, 39] and the supramarginal gyrus [37, 51], and our results evidenced several clusters of activation in the temporo-parietal areas (Fig 3). Indeed, the concept of agency arises from a combination of several multidimensional processes, including mismatch detection [34, 51], action awareness [33], or sensory-motor conflicts [37]. In particular, the role of the angular gyrus has been attributed to inter-sensory mismatch detection [27, 30, 32–34, 38, 41, 51], action awareness [33, 36, 37, 41], and integration of multisensory information [33, 37, 38, 41], often related to body ownership and SoA, whereas specific sensory-motor conflicts are processed in the supramarginal gyrus [37]. Further on the subdivision of specific aspects of the SoA, one study reported differential activation in the posterior superior temporal sulcus (pSTS), and in the middle temporal gyrus, in response to differences in the processing of perspective changes [27]. A task involving a change in perspective, mentalizing and deception (e.g., a task that requires a distinction of perspectives by assigning actions to either oneself or someone else) activates the TPJ, whereas tasks with no changes in perspectives (e.g., tasks with simple disruption of motor control) activate the pSTS. This suggests that changes in perspective contribute to the SoA, and help assigning motor representations to oneself or to an external agent. Taken together, these findings show that different neural mechanisms, with specific neural correlates, contribute to agency formation when motor control is disrupted, and that a brain area common to all of them can be found in the TPJ.

Similar considerations can be done for the lateralization of the TPJ. Several studies have found bilateral activation [21, 25, 27, 28, 33, 34, 36, 38, 39, 42, 51, 52], as confirmed by our results. However, some studies have found predominant activity in the right hemisphere [26, 30–32, 37, 41], or in the left one [3]. A clear distinction between functions of the TPJ in the two hemispheres has not been described, but it has been hypothesized that the left, rather than the right, parietal cortex plays a role in visuo-motor integration for goal directed actions [52]. However, our results evidenced a potential confound in the handedness of participants, as most of the tested subjects were right-handed, and this could generate asymmetries in the activity of the TPJ, which in turn might elicit differential neural responses across hemispheres [28]. Although it was not the focus of this meta-analysis, studies in neuropsychiatric patients have shown that lesions of the right parietal cortex are associated with patients’ feelings that their limbs do not belong to themselves [54, 55], and studies with schizophrenic patients have found an association between hyperactivity in the right parietal cortex and the feeling that their own actions are controlled by someone else [4, 56, 57]. This then suggests that agency attributions are mainly processed in the right TPJ.

### Positive agency

No significant clusters of activation were found for positive agency. Overall, the network of positive agency is not well-understood yet, and a pattern across the literature is difficult to find. One explanation that may account for this lack of consistency is that full control and positive agency are the default, with different regions and networks only becoming responsive during the loss of control [36, 58]. In line with the comparator model, when no sensory discrepancy is detected, the movement is considered self-generated, and its sensory effect is canceled.

Interestingly, it has been hypothesized that this differential brain activation can be used to distinguish between self- and externally generated movements, as only the latter elicit a neural response in relation to agency [58, 59]. This theory has found confirmation in behavioral studies, where participants rated self-administered tactile stimuli as less tickly than the same stimuli generated externally [60], as well as in fMRI studies, where neural activity was found lower when tactile stimulation was self-produced compared to when it was externally produced [61], and studies with magnetoencephalography, where responsiveness of the auditory cortex was reduced when participants spoke themselves compared to when they heard a sound played back [62].

### Other brain areas involved in the SoA

Several areas commonly associated with the SoA, such as the SMA or the insula, were not revealed by our analysis. The SMA has been found responsible for processing agency error during action execution [63, 64], and for providing predictions of the sensory consequences of an action to other brain regions, thus enabling agency formation [31, 65]. Similarly, the insula has been associated with multimodal integration of different signals related to the executed action [25, 32, 38], including emotional and visceral signals [66], and subjective timing [67, 68]. We did not find activity in these areas, and this may be due to the fact that these areas respond only to specific cognitive processes which marginally contribute to agency formation, and are not key arears for the SoA in relation to motor action. Our analysis revealed neural substrates shared across all these processes, within the TPJ.

As for explicit judgement of agency, little research has been dedicated to its neural correlates [21, 41]. Explicit assignments of agency have been correlated, for instance, with activity in dorsal fronto-median cortex [41], indicative of an interpretative, higher-level mechanism, incorporating contextual knowledge and belief reasoning. Other neural correlates of the judgement of agency have been found in the anterior prefrontal cortex and the orbito-frontal cortex [21], regions associated with self-reflective processing, receiving input from subcortical structures related to appraisal of self-relevant sensory stimuli [69], and from areas of the lateral pre-frontal cortex relevant for conscious judgements about the self [21].

Overall, the SoA seems to arise from a combination of bottom-up and top-down processes with separate neural substrates, the pre-reflective feeling of agency, relying on the comparator model, which shapes a high-order reflective judgment of agency [17, 41]. Preconscious processing is associated with activity in the TPJ, which may spread to the frontal areas when it reaches consciousness [38].

### Strengths and limitations of the current study

The main strength of our study is the use of whole-brain analysis to take into account all brain areas responsible for the SoA during motor execution. To the best of our knowledge, this is a novel approach, as previous meta-analyses focused on specific areas (regions of interests) only [21, 43], or on the SoA from a general point of view [1], and not in relation to motion, as it should be studied [44]. We thus excluded studies with, for instance, auditory [39], or pure visual [3] stimulation. Moreover, the last meta-analysis was conducted about ten years ago [1], and we could therefore include the results of the most recent studies in the field.

One potential limitation is in our definition of agency, which involved a general mismatch between motor output and visual feedback. We did not control for specific components of agency, targeted by single studies, which could have evidenced different areas, such as the SMA and the insula. However, our results evidenced that, despite specific subparts of agency, the TPJ is the area consistently active during decreased agency, strengthening the model of mismatch detection and its neural correlates. In line with this, another limitation is the focus on the feeling of agency only, which left aside the brain network of explicit judgement of agency. We decided not to include in the meta-analysis studies focusing on the judgement of agency, as our search identified only two experiments [21, 41] investigating this topic. Future neuroimaging studies should thus focus more on this aspect. Another limitation is the time window we selected, i.e., 20 years, that allowed us to include in the analysis only the most recent studies. We decided to search the literature only after 1999 because the definition of the SoA, as well as the quality of the neuroimaging techniques, may have been different earlier, and could not guarantee the inclusion of studies with comparable results. Last, our search criteria mainly relied on the keyword “agency”. We used the “related search” option to include as many studies as possible on this topic, but we cannot exclude that some studies, which defined “agency” with another keyword, were not detected by our search strategy.

## Conclusions

We performed a quantitative, whole-brain, meta-analysis of 22 neuroimaging studies on the SoA for motor control, which confirmed a clear network subtending negative agency, whose main nodes are in the bilateral TPJ (MNI: 50, -54, 14; -44, -52, 42; -48, -56, 8). Even if bilateral activation was found, the right TPJ confirmed its crucial role in detection of sensory-motor discrepancy [33]. No clear network was found for positive agency.

These results set the background for future regions-of-interest (ROIs) studies on the investigation of neural correlates of the SoA during motor control, as they provide the precise location of brain areas involved in the network of agency, as well as a neural subdivision of various aspects of agency processing related to motion execution. Our results will also serve as a background for studies aiming to use non-invasive brain stimulation techniques to modulate the SoA in healthy subjects and patients with disorders involving perturbed sense of agency (e.g., schizophrenia [4], alien hand syndrome [6], or functional neurological disorders [7]).

## Supporting information

S1 Checklist (PDF)
